# Supplementary material for: Are carbon emissions trading and green financial instruments synergistic? -Comprehensive quantitative research based on content analysis
Source: PLoS One. 2024 Mar 7;19(3):e0298601. doi: 10.1371/journal.pone.0298601 (PMC10919601; doi:10.1371/journal.pone.0298601)
Supplement: S1 Data — (ZIP) [file pone.0298601.s001.zip › Data/地级市Z_F工作报告.zip!/╝¬┴╓/╦╔╘¡╩╨/└┤╫╘╣1⁄2╓┌║┼í╛┬φ┐╦╩2╛▌═°í┐_2014.docx]

各位代表：

　　现在，我代表市人民政府，向大会作工作报告，请予审议，并请各位政协委员提出意见。

　　一、2013年工作回顾

　　过去一年，是我市发展进程中极不平凡的一年。面对经济下行压力加大的宏观形势和防汛抗洪、抗震救灾等诸多挑战与考验，在省委、省政府和市委的坚强领导下，全市各族人民紧紧围绕“转型发展、富民强市，建设幸福松原”，攻坚克难、真抓实干，取得来之不易的发展成果，实现了本届政府良好开局。

　　一年来，我们坚持稳中求进，着力推进经济健康发展。致力于夯实发展基础，结合全省“十二五”规划中期评估微调，科学调整并完成2013年主要经济指标。预计全市地区生产总值实现1650亿元，增长8%（不变价）；地方级财政收入完成62亿元，同比（下同）增长6.9%；全社会固定资产投资完成1196亿元，增长15%。致力于走内涵式发展道路，积极调整经济结构，三次产业、油与非油经济比重进一步优化。社会消费品零售总额实现512亿元，增长13%。万元GDP能耗下降2.4%。

　　一年来，我们坚持以人为本，切实保障和改善民生。城镇居民人均可支配收入和农民人均纯收入分别达到25500元和9800元，分别增长17.5%和14.5%；人均储蓄存款余额达到15810元，增长12.7%；群众生产生活条件进一步改善。面对地震、洪水两大自然灾害和严峻的安全生产形势，把捍卫人民生命财产安全作为民生之最，打赢了抗震救灾持久战、防汛抗洪保卫战和安全生产攻坚战。抗震救灾紧急疏散并妥善安置群众6.7万人，投入救灾资金1.9亿元，建设彩钢房3980所，救灾安置补贴全部发放到位，震区及周边群众余粮全部敞开收购，恢复重建规划全面启动。这次震群型地震，我们创造了“无一人重伤、无一人死亡”的奇迹。防汛抗洪众志成城、高效除险，全面战胜“两江一河”洪水侵袭。深入开展安全隐患大排查大整改，安全生产形势保持总体稳定，在全省评比中获综合一档、总分第一的好成绩。

　　一年来，主要做了以下工作：

　　（一）坚持优化升级，积极推进工业经济平稳增长。全市新增规模以上工业企业53户，规模以上工业增加值实现680亿元。主导产业加快发展。油页岩勘探开发利用示范基地建成使用，吉星日产50万立方米LNG等项目建成投产，富德20万吨EVA、吉港200万吨LNG和松原石化搬迁改造项目扎实推进。中粮健康生猪产业化一期竣工，盼盼食品、正邦200万头生猪养殖屠宰加工、齐全30万吨饲料加工、曙光3000万只肉禽屠宰等项目进展顺利。传统优势产业发展态势强劲。大唐长山热电厂1号机组投入运营，众合热电联产试运行，国能生物质发电、优能风电等项目快速推进。中化长山改扩建稳步实施，苏源50万吨复合肥投入生产。五环汽车模具、威龙金属加工等项目顺利实施。鹏鸿木业地板等项目开工建设。尼普洛嘉恒医药包装等项目建成投产。创新发展能力不断增强。经开区晋升为国家级经济技术开发区。荣亿石油设备等4户企业被认定为省高新技术企业。大多陶瓷内衬管产业化被省政府确定为重大科技成果转化项目。松岭汽车零部件、隆运达水泥等技改项目稳步推进。中化长山氮肥工程技术研究中心投入运营，前进石油机械等3户企业被认定为省级技术中心。

　　（二）坚持增产提效，加快发展现代农业。农业综合生产能力大幅提升。哈达山水利枢纽工程基本完工，松原灌区工程前期工作取得阶段性成果，河湖连通工程启动实施。整理土地40.7万亩。粮食生产实现历史性突破，总产量达到750.3万吨。现代农业发展水平显著提高。农业高新区现代农业技术试验示范被纳入中科院东北农业创新集群发展战略，哈达山生态农业旅游示范区跨入省级开发区行列。前郭灌区红光农场省级院士工作站设立。建设高光效示范区30万亩，创建粮油高产示范片95个，完成玉米膜下滴灌82.4万亩。新一代天气雷达预警业务中心楼主体完工。农业产业化步伐加快。新增省级龙头企业4户、专业合作社704个、土地规模经营面积25.8万亩。棚菜种植面积突破10万亩。“查干湖大米”被评为吉林省名牌产品。新建标准化规模养殖场（小区）156个。实现畜牧业增加值75.7亿元，增长6%。

　　（三）坚持多点发力，大力推进服务业提档升级。服务业增速继续位居全省第一。突出发展现代商贸物流业。哈萨尔购物广场、红星美凯龙家居生活广场投入运营，蓝湾都汇、哈剌和林国际城一期主体完工，东镇城市广场、沃尔玛超市开工建设。松西物流及内陆港投入运营，雅达虹煤炭医药物流园区建设快速推进。健康发展房地产业。开发房地产220万平方米，增长81%；销售商品房116.4万平方米，增长26%。大力发展特色旅游业。哈达山景区晋升为国家级水利风景区。辽帝春捺钵遗址群被确定为全国重点文物保护单位。实现旅游综合收入57亿元，增长24.3%。积极发展金融业。引进保险公司3家，新增小额贷款公司11家。创新质保通等金融产品21种。油田配套和小油田企业账款质押、顺位抵押等融资模式有效实施。金融机构贷款增量达到126.6亿元，存贷比提高11.1个百分点。

　　（四）坚持联动发展，同步推进城乡建设。中心城市建设呈现新气象。完成《松原市城市总体规划》修编和城市给排水等17个专项规划。投入资金45亿元，实施重大城市基础设施项目50个。“一江两岸”建设扎实推进，生态环保型壅水坝主体竣工，天河大桥开工建设；完成沿江东路、松原大路、东镇大路、哈达大街等13条街路新建、续建或改造；苏力德公园建成使用，成吉思汗文化园对外开放；松花江大桥等4处重点市政工程和120座临江楼宇全面亮化。完成城区29条街路和35个公园、广场及绿岛绿化，新增城市绿地20万平方米，街路绿带实现全覆盖。中小城市和新农村建设步伐加快。完成扶余撤县设市。长山镇入选全省首批特色试点镇。新建省级新农村示范村（农场）120个、新型农村示范社区50个。综合交通运输体系进一步完善。全面完成松原客运交通枢纽与站前综合改造、松原机场项目前期工作。松陶铁路建设进展顺利。绕城高速竣工通车，我市成为全省首个拥有环城高速的地级城市。江南公交调度中心建成使用。省道304线前七号至科左中旗段二级路续建、瓦房街公铁立交桥等工程竣工通车。新建农村公路276.8公里，全市行政村全部实现晴雨通车。

　　（五）坚持改革开放，着力激发经济社会发展活力。重点领域改革稳步推进。新一轮政府机构改革正式启动。取消和暂停行政审批项目17个、收费项目14个，审批时限平均压缩20%。事业单位分类改革有序推进。营改增试点启动实施。积极开展综合治税、税费清查和总部经济培育，增缴税费5.7亿元。厂办大集体改革全面启动。基本药物零差率销售和县级公立医院改革试点稳步开展。民营经济快速发展。新增民营企业1166户、个体工商户1.6万户。德源智能等6家公司晋升为省级民营科技企业。发展吉林省著名商标26件。民营经济主营业务收入实现1848亿元，增长17.8%。招商引资成效显著。成功引进沈铁、大商等战略投资者。曙光生物有机肥、蒙牛荷斯坦奶牛养殖及高端奶制品加工等大项目成功签约。招商引资到位资金805亿元，增长15%。

　　（六）坚持成果共享，统筹推进民生和社会事业发展。大力实施民生行动计划。新增城镇就业5.9万人，转移农村剩余劳动力55.3万人。新增城乡养老保险1万人，为11万参加城保退休人员增发养老金2.3亿元。医疗保险实现市级统筹。新农合住院补助封顶线提高到10万元。完成城乡低保提标。发放救灾救济资金2159万元。改造棚户区70万平方米，综合整治D级危房15万平方米、老旧小区108万平方米，完成暖房子工程100万平方米，建设分配廉租房5万平方米，改造农村危房7000户。发放住房公积金贷款4.5亿元。改造中心城区背街巷路45条。新建扩建热源厂3座，新建改造供热管网59公里，增加供热能力430万平方米。铺设天然气管网10公里，新增燃气管道用户3.2万户。江南新水厂投入使用，新建大型二次供水站9座，改造小型二次供水站70座，实现城区供水能力与饮水质量双提升。有效解决9万农村人口饮水安全问题。改造农贸市场2个。建成食品安全快速检测站119个、低保特惠商店2个。新开通市区近郊城乡公交线路3条，实现城区居民刷卡乘坐公交车。年初承诺的93件民生实事，除2件因宏观政策调整或影响城市空间布局而取消，其余全部完成年度任务。全面发展社会事业。油田十二中新教学楼、逸夫小学综合楼落成使用，建成市级示范性中小学、优秀村级小学各20所，改造薄弱学校30所，新改扩建公办幼儿园4所。市中西医结合医院投入使用。免费实施白内障复明手术1069例。市残疾人康复中心续建完工，市脑瘫儿康复基地建成使用。新建农村文化大院473个，农民自办文化示范项目通过国家验收评审，满族新城戏传承保护中心被评为全国地方戏创作演出重点院团。新增农村有线电视用户2300户。市体育馆主体封闭。成功承办第六届全省农民运动会。

　　各位代表！一年来，我们切实加强社会治理。扎实开展“六五”普法。圆满完成第九届村民委员会换届。突出解决群众反映强烈的热点难点问题，实现全国“两会”等重要敏感时段“零非访、零登记”。着力推进平安松原建设，妥善处置各类突发事件，群众安全感、满意度持续提升。一年来，我们切实加强政府自身建设。自觉接受人大的法律监督、工作监督和政协的民主监督，办理人大代表建议66件、政协提案207件。坚决贯彻中央“八项规定”，深入开展机关作风集中整顿活动，大力优化经济发展软环境，机关干部服务意识和行政效能进一步提高。此外，民族宗教、外事侨务、双拥共建、统计、审计、档案、质监、人防、地方志、红十字等工作都取得了新成绩。

　　各位代表！过去一年，我们沉着应对、科学施策，战胜了重大挑战，经受住了严峻考验，实现了保增长、惠民生、维稳定目标任务。回顾一年的工作，我们深感成绩来之不易！这是省委、省政府和市委统揽全局、正确领导的结果，这是市人大和市政协有效监督、大力支持的结果，这是全市人民凝心聚力、砥砺奋进的结果，这是中省直单位、驻松部队、武警官兵和社会各界共克时艰、团结共建的结果！在此，我代表市人民政府，向所有为松原发展建设作出贡献的同志们、朋友们，表示衷心的感谢！

　　二、准确把握我市发展面临的总体形势

　　当前，松原经济社会发展已经步入结构调整阵痛期，正处于倒逼转型、爬坡过坎的重要历史拐点。困难之多、压力之大，前所未有。要科学谋划和部署2014年工作，必须准确把握这一阶段性特征，切实增强紧迫感和使命感，克难奋进，积极而为，努力实现全市经济社会更高质量、更具活力、更富效率、更可持续发展。

　　新的一年，我们要清醒面对困难和问题。从产业结构看，松原作为典型的资源型城市，“一油独大”的产业格局还没有实现根本转变，特别是受原油量价齐跌影响，长期积累的深层次结构性矛盾日益凸显。从发展质量看，非油经济和地方工业增长乏力，龙头企业和知名品牌较少，实体经济呈现“转虚”倾向，财政收入占GDP比重明显偏低。从增长后劲看，油气资源红利递减，资源占有和转化空间受限，经济发展上行因素与资源硬约束相对冲；能够拉动投资的大项目、支撑跨越的大企业偏少，新的经济增长点不多，经济增长内生动力不足；生态环境修复成本加大。同时，改善民生仍然任重道远，安全生产形势依然严峻，软环境、行政效能与加快发展的需要还存在差距。对此，必须认真加以解决。

　　新的一年，我们要倍加珍惜发展机遇。从宏观经济环境看，国家新一轮改革力度空前，稳增长、调结构、惠民生政策密集出台，改革红利加速释放，我市被国务院列为成长型资源城市和生物质能源产业集群城市，吉林省西部生态经济区开发战略深入实施，为我们加快发展提供了难得历史机遇。从转型发展条件看，未来几年，中石油公司将与民企合作，投资156亿元，开发松原矿区27个未动用含油区块，新增产能133万吨，为我市接续产业发展赢得宝贵缓冲期。页岩气开发上升为国家战略，我市已探明储量油页岩可提炼约30亿吨页岩油，将成为新的经济增长极，为转型发展再注强劲动力。从松原自身实际看，近年来开工建设的一批重大项目从今年起陆续投产达效，将成为拉动我市经济发展的新亮点。哈达山水利枢纽工程全面运行，松原灌区等河湖连通工程启动实施，将拉动我市农业和畜牧业经济快速增长。全市上下人心思进，谋转型、求发展的愿望更加强烈而迫切，必将转化为赶超晋位的强大正能量。只要我们乘势而上、团结奋进，就一定能够战胜困难、赢得未来！

　　新的一年，我们要勇于担当阔步前行。面对新的形势和任务，必须解放思想、敢于突破，在改革中开拓，在进取中作为；必须急而不躁、脚踏实地，一步一个脚印儿，真干实干快干；必须坚定信心、奋力攻坚，在结构调整上求提速、在产业发展上求升级、在项目建设上求加力，从而牢牢把握发展主动权，将美好蓝图变为生动现实！

　　三、2014年主要工作

　　2014年，是我市完成“十二五”规划和全面建成小康社会关键之年。做好今年政府工作，意义尤为重大。政府工作的总体要求是：全面贯彻落实党的十八届三中全会和省委市委全会精神，以“转型发展、富民强市，建设幸福松原”统领全市经济社会发展，紧紧围绕实施“美丽家园”行动计划，以改革创新为着力点，以招商引资和项目建设为突破口，更加注重抓统筹、可持续，更加注重转方式、调结构，更加注重解难题、破瓶颈，更加注重保民生、促和谐，着力打造松原经济“升级版”，努力开创松原发展新局面。

　　按照这个总体要求，2014年全市经济社会发展主要预期目标是：地区生产总值增长8.5%左右，地方级财政收入与经济增长同步，全社会固定资产投资增长15%以上，社会消费品零售总额增长11.3%，城镇居民人均可支配收入和农民人均纯收入分别增长10%和11.2%，城镇登记失业率控制在4%以内，万元GDP能耗下降2.2%。

　　实现上述目标任务，全年要做到“三个突出”：一是突出科学发展。坚持稳中求进、综合提质，使经济增长既保持在合理区间，又不断提高质量和效益。二是突出“美丽家园”建设。以“六个专项行动”为重点，突出绿色发展理念和生态文明建设，扎实推进松原特色城镇化，进一步提高城乡综合承载力和秀美宜居度。三是突出民生改善。继续实施民生行动计划，从百姓最关心、最需要、最期盼的实事做起，切实解决涉及群众利益的突出问题，不断提升全市人民幸福指数。具体做好八个方面重点工作：

　　（一）围绕招商引资上项目，推动经济规模加快壮大。突出项目建设这个重点，打好招商引资“组合拳”，千方百计引进一批战略投资者，集中精力布局和建设一批重大优质项目。好中求快建项目。全年开复工3000万元以上项目500个，其中亿元以上项目70个。推动中科田园产业园、长青10万吨成型燃料等项目投产达效，完成北大荒食用油等项目扩能，加快推进中化长山“36-60”扩能改造，开工建设鹰龙彩印覆膜包装材料、三和鼎晶瓷、济润源软体沙发及实木家具等重点项目。多中求优引项目。围绕发挥资源优势，争取光禾非粮生物质能源、金洋玉米秸秆综合加工、万兴6万吨花生油、翔润蓖麻油深加工、华能集团光伏地热发电等项目落地开工。围绕发展加工制造业，争取奥凯公司种子机械设备生产、德力集团玻璃器皿加工等项目签约落地。围绕延伸产业链条，依托盼盼食品、嘉吉生化等企业，积极引进下游项目，提高粮食加工产业丰厚度。全年招商引资到位资金850亿元。实中求精谋项目。准确把握国家和省政策导向，立足我市资源优势和产业基础，谋划包装60个重大产业项目、25个重大基础设施项目、15个重大民生项目。

　　（二）围绕提质量促增效，推动工业经济发展壮大。坚持规模化和多元化发展方向，加快推进结构调整和产业升级，增强工业经济实力和抗风险能力。新增规模以上工业企业20户；规模以上工业增加值实现740亿元，增长8.8%。促进油气经济巩固壮大。全力支持吉林油田和东北油气两大企业稳油增气，积极推进27个未动用含油区块开发，争取油气产量当量达到700万吨。开工建设艾克石油钻探项目。加快推进吉港200万吨LNG、富德20万吨EVA、松原石化搬迁改造等大项目建设，重启四氢呋喃项目，拉长油气化工产业链条。促进工业结构不断优化。着力发展农产品加工和食品业。推动盼盼食品、齐全30万吨饲料加工等大项目建成投产，加快推进中粮健康生猪产业化、正邦200万头生猪养殖屠宰加工等项目建设，开工建设吉林交通集团食品加工项目。着力提升装备制造业。促进阳光汽车部件项目建成投产，开工建设天泽汽车内饰项目，积极帮助为油田配套的地方企业争取更多份额。着力发展能源产业。争取长山热电厂9号机组延缓关停，加快推进2号机组建设前期工作。推动聚太生物质热电联产等项目竣工投产。开工建设亿沃福太阳能光伏发电项目。着力培育化肥和生物制药产业。开工建设史丹利复合肥项目，推动百年汉克制药等项目投产达效。促进工业企业发展升级。加快培育绿色食品、清洁能源等低碳环保型产业，探索绿色发展道路。帮扶停产、半停产企业尽快复产，逐步实现健康发展。引入现代经营模式，鼓励开展产学研联合，推动企业不断加强科技和管理创新。

　　（三）围绕抓集约增效益，推动现代农业快速发展。抓住我省规划建设西部生态经济区机遇，大力发展特色高效农业，进一步提高农业经济贡献度。实现增加值285亿元，增长7.5%。提升农业综合生产能力。哈达山水利枢纽工程竣工。实施河湖连通推进行动，开工建设松原灌区和引松入扶工程，争取完成查干湖补水工程。开发整理土地13.6万亩，新建高标准基本农田10.5万亩。加快国家级节水示范区建设，完成玉米膜下滴灌78万亩。正常年景全市粮食总产量达到800万吨。推进农业产业化经营。强化松粮集团拉动作用，着力提升“查干湖”品牌知名度。推广长岭云天化农业合作社、农业高新区基地合作社等集约经营模式，引导承包经营权有序向专业大户、家庭农场、农业企业流转，发展多种形式规模经营。新增市级以上农业产业化重点龙头企业10户、土地规模经营面积37.5万亩。新增棚菜面积0.7万亩。无公害、绿色、有机作物种植面积发展到150万亩。加快牧业强市建设。推行生态牧业发展模式，发展牧业循环经济。推进标准化、规模化、产业化经营，新增标准化规模养殖场（小区）160个。实现增加值80.3亿元，增长7%。

　　（四）围绕做特色提档次，推动现代服务业扩量升级。坚持挖掘潜力、培育增量，把服务业打造成经济发展新引擎。实现增加值665亿元，增长8.1%。促进旅游业整合发展。启动全市旅游总体规划修编，完成查干湖国际旅游经济区等规划编制。突出生态和民族特色，谋划推进查干湖、泥林、龙华、天河谷温泉、哈达山、三江口湿地、大金碑等景区景点综合开发，构建松原特色旅游线路。启动妙因寺扩建工程。办好查干湖冬捕活动。实现旅游综合收入68亿元，增长19.3%。促进商贸物流业升级发展。加快东镇、欧亚等商圈建设。蓝湾都汇、松原汽车产业园建成运营。完成前郭中心市场升级改造。继续推进“万村千乡市场”工程。争取香港豪德城市商贸物流园、苏宁城市生活广场、万达城市综合体开工建设。拓展对外贸易合作，进出口总额实现1亿美元，增长10%。促进房地产业健康发展。合理调控开发节奏，建立科学供地机制。区别建立各类住房供应体系，激活商品房刚需市场。开发房地产270万平方米。促进金融等新型业态加快发展。争取光大、兴业等银行在松设立分支机构，新增小额贷款公司5家。规划建设春捺钵遗址园和辽金特色文化产业园，争取辽金文化城晋升为省级文化产业示范基地。积极发展会展、休闲、社区服务等新兴产业。加快培育总部经济。

　　（五）围绕育增量强实力，推动板块经济加速壮大。做强县域经济。助推各县（市）科学谋划城市和产业发展定位，加大招商引资和承接产业转移力度，促进各类园区整合升级，引导相关产业和企业向园区集聚，加快培育特色优势和战略性新兴产业。大力发展现代农业。打造县域城市综合体和跨县（市）旅游产业联盟。实施扩权强镇战略，着力扶持中心镇、重点镇和节点镇。探索建立县级农村产权交易平台和以林权、草场权等收益为保证的融资模式，扩大土地收益保证贷款试点。完善绩效考核体系，对地方级财政增幅较大的县（市）给予奖励。做优开发区经济。突出主导产业地位，引导产业错位发展，加快将经开、石化、查干湖、农业高新和哈达山五个开发区分别打造成食品加工与油田配套、油气化工、特色旅游、现代农业的产业航母，争取扶余工业集中区晋升为省级开发区。探索采取加大投资强度、统筹摆放项目、实行财税分成等措施，促进产业加速集群，尽快形成乘数效应。建设高效融资平台，加强路网、管网等基础设施建设。突出招商引资主力军作用和项目建设主战场功能，进一步增强开发区综合吸引力和辐射带动力，力争各开发区分别引进3个10亿元以上项目。做大民营经济。全面落实国家和省市支持小微企业发展政策。继续开展包保民企大走访活动。对支持民营企业发展贡献大的金融机构给予奖励，将市中小企业担保公司担保能力扩大到25亿元，多渠道缓解民营企业融资之困。鼓励民营企业争创驰著名商标。建立雅达虹中小企业创业孵化基地，免费开展创业者、小老板和企业家培训，助推民营经济加速腾飞。

　　（六）围绕强功能塑形象，推动城乡建设加快进程。按照以江为轴、两岸并举、组团发展、城乡一体的原则，强化规划引领、功能完善和品位提升，努力提升城乡建设水平。完善城乡规划体系。编制《松原市统筹推进城镇化总体发展规划》和城市排水防涝、城市抗震工程等专项规划。完成中心城区61平方公里控制性详规编制。推进“一江两岸”建设。实施滨江大道延伸、前程大路续建工程，完成生态环保型壅水坝建设、天河大桥年度建设任务和巴尔达等公园续建，开工建设天河岛湿地公园；完成东镇大路、纳仁汗等道路和公园绿化升级；完善提升江心岛、堤坝及两岸道路亮化；塑造松花江城区段水秀景观，进一步彰显中心城市的滨江特色、生态品质和人文内涵。提升城市综合承载功能。加大棚户区和江北老城区改造力度。实施和平路、松原大路、沿江东路、前炼南路等道路延伸、新建或续建和阿阑豁阿大街下穿铁路立交桥等建设工程。完成乌兰大街、长宁街等11条主街路综合改造。开工建设松原机场。实施松原客运交通枢纽与站前综合改造工程，加快实现松原火车站与客运站之间“零换乘”。松陶铁路竣工通车。争取完成长白铁路扩能改造征地拆迁。完成宁江客运站主体工程建设。开工建设省道哈松公路后河拉林至宁江段。完成“数字松原”地理空间框架建设。争取二期15亿元企业债券等融资全部到位。加快中小城市和镇村建设。推进扶余、长岭、乾安和特色小城镇建设，促进产业与城镇深度融合。深入推进“千村示范、万村提升”工程。完善建设新型农村示范社区50个。新建农村公路70公里。抓好地震灾区恢复重建。结合新农村建设和农村危房改造等重点工程，坚持高起点、高标准规划，统筹推进倒毁民居和学校、医疗卫生机构等公共服务设施重建，加强指导、服务和监督，严把设计、材料和施工质量关，提高新建房屋抗震等级，国庆节前全面完成重建任务。

　　（七）围绕惠民生增福祉，推动群众生活持续改善。重点做好八个方面民生工作。社会保障方面。发放小额担保贷款5000万元，新增城镇就业4.5万人，着力帮助特殊群体就业，保持城镇“零就业家庭”动态为零，转移农村剩余劳动力50万人。新增城镇职工基本养老保险和城居保、新农保参保1.5万人，进一步扩大城镇居民基本医疗、工伤、失业、生育保险及新农合覆盖面，继续提高城乡低保补助标准。市残疾人托养中心基本完工，市精神病院综合楼建成使用，启动政府购买居家养老服务试点。安居保障方面。改造棚户区97万平方米，建设廉租房16.9万平方米，实施暖房子工程75万平方米，改造农村危房1.5万户，解决720户历史遗留回迁户安置问题，完成无籍房产权产籍发放。市政公用建设方面。新建松江热源厂，改造老旧供热管网92公里。新建改造天然气管网32.7公里。改造升级江南、江北两个水厂引水工艺，完成市城区二次供水站改造和江北净水厂哈达山取水管线工程。启动县（市）及具备条件重点镇“放心水”工程，争取再解决5万农村人口饮水安全问题。新建220千伏环网网架。建设弱电管网32.4公里。实施市区内公交车“暖车厢”工程和刷卡乘坐出租车试点，新建30座高档公交候车亭。背街小巷改造方面。改造民珠胡同、市政街、爱珂东路等25条巷路，有效改善背街小巷附近居民生活环境。社区建设管理方面。综合整治老旧小区100万平方米，加强对183栋单体楼、弃管楼及已改造老旧小区综合管理，进一步完善社区服务功能。文教体卫惠民方面。举办全市首届社区文化艺术节和第二届农民文化艺术节，组织放映电影1.34万场。新建市级示范中小学19所、优秀村级小学20所，新改扩建公办幼儿园10所，开工建设市第二高中和油田第十一中学综合教学楼。市体育馆投入使用。巩固完善基本药物制度，深入推进县级公立医院改革。异地新建市中心医院，开工建设市儿童医院，临江社区卫生服务中心建成使用，加快标准化村卫生室建设。继续开展好国家免费优生健康检查。平安松原建设方面。实施城区53个交通路口路段渠化项目。农村视频监控系统覆盖面达到50%。完成活断层探测与地震危险性评价项目建设，市地震前兆动植物观测站投入使用。建设江南民政救灾物资储备大库。环境综合改造方面。坚持“先地下后地上”原则，强化城区积涝综合治理，新建改建泵站10座，加快实现雨污分流。完成江南、江北污水深度处理及污泥干化项目建设，开工建设石化园区污水处理厂。推进生活垃圾焚烧发电项目建设，新建水冲公厕和垃圾压缩转运站15座。建设城市空气质量自动监测站两座，实施机动车尾气治理工程。推进市容环境卫生整治，进一步净化美化城市。实施农村环境提升和城乡绿化建设行动，完成“四清”“四改”村300个，启动环城绿化带、城镇绿化、绿色通道等工程建设，实施30个村屯道路绿化工程，完成人工造林3.8万亩、义务植树263万株，新农村示范村绿化率达到35%以上。

　　（八）围绕维稳定促和谐，推动社会治理不断创新。适应社会转型需要，不断提高治理科学化水平，确保社会既充满活力又和谐稳定。强化社会综合治理。深入开展“六五”普法。强化对流动人口和特殊人群管理服务。依法加强宗教管理。畅通信访诉求渠道，实行网上受理信访制度，健全及时就地解决群众合理诉求机制，建立涉法涉诉信访依法终结制度，全市初信初访办结率达到95%以上。加强网络虚拟社会监管，营造健康文明的网络环境。强化安全生产治理。坚持管行业必须管安全，深入推进安全生产标准化达标和网格化、社会化监管，继续加大重点行业和领域隐患排查整改力度，严厉打击各类违法违规生产行为，坚决遏制重特大事故发生。强化公共安全治理。全面加强社会治安，严密防范、严厉打击各类违法犯罪活动。扎实推进食品药品安全放心工程，保障群众饮食用药安全。加强突发事件应急管理，不断提高突发事件预测预警和应急处置能力，有效降低各类灾害、事故造成的损失。

　　各位代表！全面完成今年各项目标任务，必须根据中央顶层设计和省委省政府、市委指导意见，结合松原实际，抓好全面深化改革落实推进，深入开展政府机构和行政审批制度改革，进一步转变政府职能，以创新的思路和办法，破除发展障碍，汇聚发展能量，增添发展活力，打造松原发展竞争新优势。必须主动接受市人大法律监督和工作监督，自觉接受市政协民主监督，广泛听取各界意见建议。必须切实加强政府自身建设，不断强化机关作风建设和绩效管理，整治慵懒散，“亮剑”低效能，坚决杜绝政令执行“堰塞湖”现象，强力推进管理创新、结果问效和行政问责，不断增强政府公信力和执行力。深入开展政行风评议，严厉整治不作为和乱作为等突出问题，严肃查处涉软案件，进一步改善经济发展软环境。必须严格执行中央“八项规定”，坚决纠正“四风一顽症”，从严控制机构编制和“三公”经费支出，严禁新建政府性楼堂馆所，建立办公用房集中统一管理长效机制，及时推进公务用车制度改革。认真落实党风廉政建设责任制，加强对重要领域、关键岗位的行政监察和审计监督，着力建设从严治政的清廉政府。

　　各位代表！历史赋予重任，人民寄予厚望。站在新的起点，转型发展的使命激励着我们，爬坡过坎的挑战考验着我们，全市人民过上幸福生活的新期待鞭策着我们！让我们在市委的坚强领导下，励精图治，埋头苦干，为创造松原更加美好的明天而努力奋斗！
